# Post-pneumonectomy Cardiac Herniation Management: A Rare Case

**DOI:** 10.7759/cureus.90288

**Published:** 2025-08-17

**Authors:** Noor ul Huda, Sadia Ali, Ahsun Khan, Saad Ur Rehman, Muhammad Faisal

**Affiliations:** 1 Anesthesiology, Shaukat Khanum Memorial Cancer Hospital and Research Centre, Lahore, PAK; 2 Anesthesiology and Critical Care, Shaukat Khanum Memorial Cancer Hospital and Research Centre, Lahore, PAK

**Keywords:** cardiac herniation, dextrocardia, lung cancer, lung-sparing resection, pneumonectomy

## Abstract

Cardiac herniation is an uncommon complication of pneumonectomy. It usually occurs when a defect is made in the pericardium during the surgical resection of the lung. It is a life-threatening complication; even after surgical correction, morbidity and mortality rates remain high. We present a case of right-sided cardiac herniation after extrapleural pneumonectomy. This case report describes the difficulty in diagnosing and managing this rare complication. It discusses the causes and pathophysiology of cardiac herniation. We have described the clinical presentation and tools to diagnose such cases. In this case report, we have discussed the available treatment options and measures to prevent this complication. In any patient who acutely deteriorates post-pneumonectomy, cardiac herniation must be considered as one of the differential diagnoses.

## Introduction

Lung malignancy is among the top most causes of cancer-related death, and to treat this problem, each year, more than 25,000 procedures are performed [1]. Surgical intervention still remains the fundamental treatment option for both early and advanced stages of lung cancer. Complete or partial resection of a lung tumor totally depends upon the grade and stage of the tumor. Unfortunately, most of the patients present at a later stage of the disease, and only 25-30% are diagnosed at an early stage; additionally, only 25-30% of patients are considered suitable for curative surgical resection [2].

Pneumonectomy is an invasive procedure that involves the surgical removal of the entire lung and is considered a management option for patients with advanced lung disease. Among all the major lung resections, it accounts for 7.5% of the cases [3]. Pneumonectomy is commonly performed for lung cancers, which are locally advanced, central tumors with invasion of vascular and/or bronchial structures [4,5]. The five-year survival rate after pneumonectomy for lung cancer depends on the stage of the disease; it may be as high as 44% for stage I and 37.5% for stage II and may be as low as 29% for stage III [6-8].

Evarts A. Graham performed the first pneumonectomy in 1933 for lung malignancy. The patient had a long survival period after the surgery. Extrapleural pneumonectomy was first described by Sarot in 1949. The procedure was initially performed in patients with tuberculous empyema, but later on, it was used for the treatment of advanced lung cancers [9].

Types of pneumonectomy include standard and extrapleural pneumonectomy. In standard pneumonectomy, the entire lung is removed, whereas in extrapleural pneumonectomy, in addition to the lung, there is an expanded resection of parietal and visceral pleura, pericardium, ipsilateral hemidiaphragm, and mediastinal lymph nodes [10]. It is mostly performed on patients with advanced malignant lung disease. Radiation and chemotherapy usually accompany the surgical intervention to improve survival in extrapleural pneumonectomy.

Pneumonectomy is associated with numerous pathophysiological changes that can adversely affect the patient's outcomes. It is associated with a large number of known complications that can occur during or after the procedure. Pulmonary function tests show a decrease in forced expiratory volume (FEV1), forced vital capacity (FVC), diffusing capacity of the lung for carbon monoxide (DLCO), and lung compliance, whereas airway resistance increases [10]. Chest X-ray following pneumonectomy usually shows that the space is initially filled with air; eventually, fluid replaces the air at a rate of 1-2 intercostal spaces per day. There is an elevation in the ipsilateral diaphragm as well as a gradual shift of the mediastinum towards the operative side [11].

Regarding cardiac function, there is an increase in resting heart rate as well as a decrease in stroke volume. There is no change seen in pulmonary artery pressure (PAP), pulmonary vascular resistance (PVR), and central venous pressure (CVP). The altered position of the heart may explain the compromised cardiac function in long-term survivors [12].

The most common complication post-pneumonectomy includes atrial dysrhythmia, which commonly occurs three days after the surgery [13]. Respiratory complications such as pneumonia, atelectasis, and acute respiratory distress syndrome are also very common. Bronchopleural fistula occurs in about 1.5-4.5% of patients who undergo pneumonectomy [14]. Post-right pneumonectomy cardiac herniation usually presents within the first 24 hours and can present with hemodynamic collapse that requires immediate surgical correction. In many cases, the orientation of cardiac chambers remains unchanged, but in severe cases following pneumonectomy, the heart may rotate at 60-90 degrees counterclockwise (levorotation) [15].

## Case presentation

A 41-year-old man with no known comorbidities presented to us through the walk-in clinic with a history of hemoptysis and weight loss. His computed tomography (CT) scan of the chest revealed right upper lobe bronchogenic carcinoma (adenocarcinoma). His case was discussed in the hospital's multidisciplinary team (MDT), and surgical resection was recommended. The surgeon decided to perform a right-sided pneumonectomy. On preoperative anesthesia evaluation, the patient had no significant medical or family history, and his examination and investigations were unremarkable with a preoperative FEV1 of 1.5 liters (normal FEV1 3-4.5 liters for young adults and 2-3.5 liters for older adults).

Sign-in was done as per the World Health Organization (WHO) checklist. Standard American Society of Anesthesiologists (ASA) monitoring was attached. A thoracic epidural was inserted at the T5-T6 level under sterile measures. General anesthesia was induced with IV midazolam and propofol. Atracurium was used for paralysis. Airway was secured with a 37Fr left-sided double-lumen tube. The left radial arterial line was secured for continuous invasive monitoring. A central venous catheter was inserted in the right internal jugular vein under ultrasonic guidance. The patient underwent right thoracotomy and right extrapleural pneumonectomy. His intraoperative findings included a lung mass with involvement of the parietal pleura, pericardium, and phrenic nerve. During the intraoperative course, the patient required boluses of phenylephrine to maintain a mean arterial pressure (MAP) above 65 mmHg. The total duration of the procedure was five hours with an intake of 3 liters of Ringer's lactate and 2 units of packed red blood cells. Urine output was 100 milliliters, and blood loss was 500 milliliters. A chest drain was inserted, and suction was not attached as per the standard protocol.

At the end of the procedure, the patient developed ST-segment elevation, which settled after raising the diastolic pressure above 70 mmHg. Once stable, awake extubation was done in the operating room (OR), and the patient was shifted to the post-anesthesia care unit (PACU) on 3 liters of oxygen via nasal cannula, maintaining a saturation of 99%. His electrocardiogram (ECG) showed T-wave inversion in leads I, II, V5, and V6. In addition to that, his serial troponins were raised as well (Table 1).

**Table 1:** Serial troponin levels

Lab test	Normal value	First value	Second value
Troponin I	Less than 0.04 ng/ml	1.42 ng/ml	1.71 ng/ml

**Figure 1:**
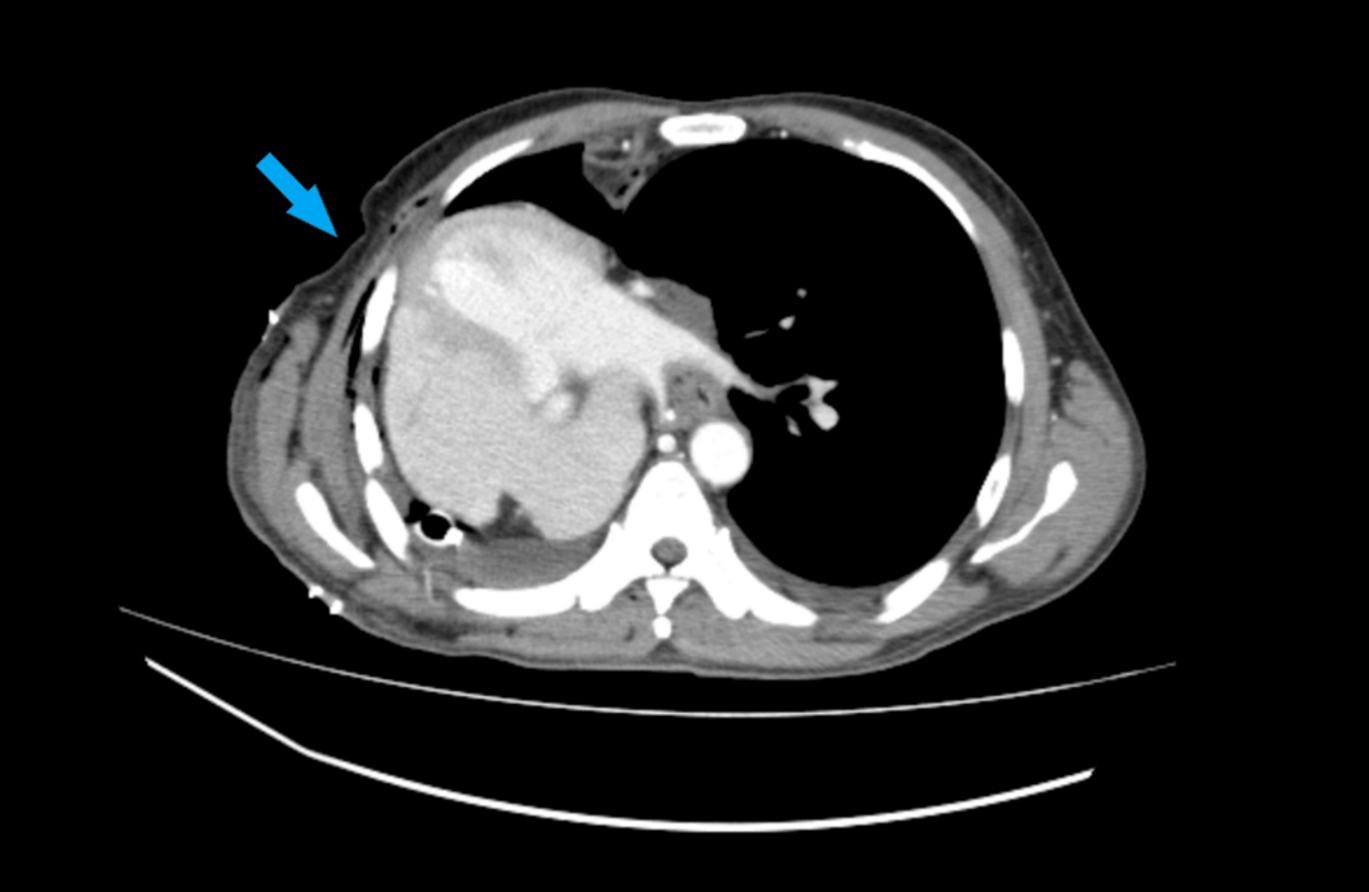
Axial contrast-enhanced CT image of the thorax Axial contrast-enhanced CT image of the thorax at the level of the left ventricle demonstrating complete right mediastinal shift (blue arrow), with the left ventricular apex facing laterally. The right atrium and right ventricle are located posteriorly, suggesting up to 180-degree axial cardiac rotation on its axis. Small bilateral pleural effusions, right chest tube, and pulmonary veins are also visualized. CT: computed tomography

**Figure 2:**
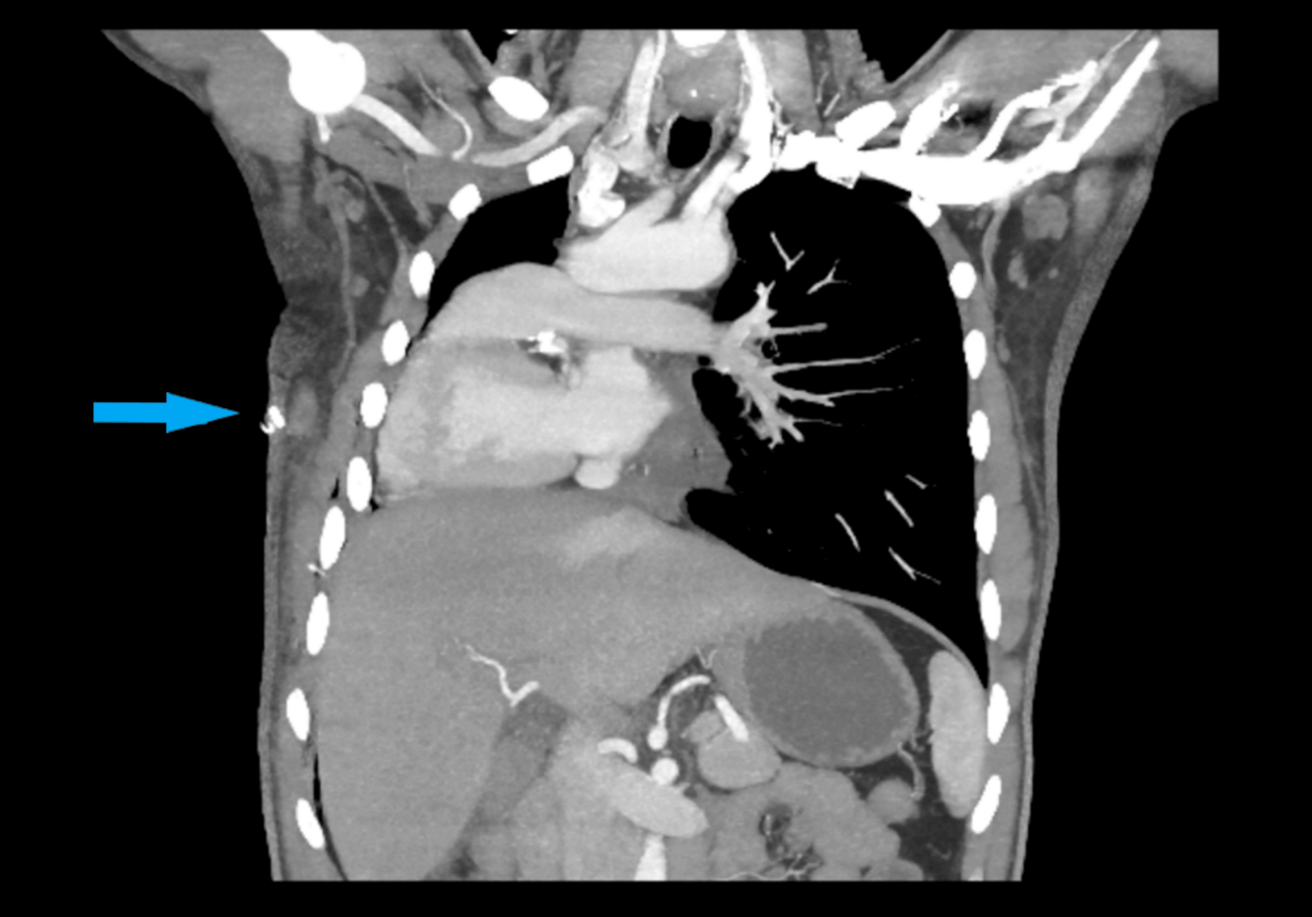
Coronal reformatted contrast-enhanced CT image of the thorax Coronal reformatted contrast-enhanced CT image of the thorax showing complete dextrocardia (blue arrow) with normal anatomical position of the aortic arch and great vessels. The pulmonary trunk is shown between the left ventricle and aortic arch. CT: computed tomography

Postoperative chest radiograph in the PACU showed a right-sided cardiac mass. This unusual finding was further investigated with a CT of the chest, which showed complete mediastinal shift towards the right, cardiac herniation in the right hemithorax with rotation on its axis, and mild superior vena cava twist with normal heart and aorta (Figures 1-2).

The patient remained stable on his first postoperative day in the intensive care unit (ICU) with low-dose noradrenaline (2-4 mcg/kg/min). On the second day, he had sudden syncope with a low MAP of 30 mmHg. The low MAP was managed with intravenous fluid boluses. The surgical team reviewed and planned for an emergency pericardium reconstruction with mesh. 

The patient was transferred from the ICU to the OR, where a slow intravenous induction was performed with Inj. Glycopyrrolate 200 mcg, Inj. Midazolam 5 mg, Inj. Propofol 20 mg, and Inj. Atracurium 40 mg. He was intubated in the left lateral position with an 8.0 endotracheal tube. Then an arterial line was placed for invasive blood pressure monitoring. During the intraoperative course, hemodynamic stability was maintained by giving frequent adrenaline and phenylephrine boluses. The significant intraoperative finding was a dilated right ventricle. The heart was repositioned into the pericardial cavity, and reconstruction of the defect was done with composite mesh.

At the end of the procedure, arterial blood gases showed respiratory acidosis (Table 2); therefore, he was transferred to the ICU on mechanical ventilation and inotropic support (adrenaline and phenylephrine). The patient seemed to be in right heart failure with no superior vena cava or inferior vena cava obstruction. Therefore, it was decided to start milrinone infusion in the ICU. Continuous boluses of phenylephrine were given along with adrenaline infusion to maintain MAP and heart rate, but there were drastic blood pressure variations and tachycardia up to 160/min during shifting. Unfortunately, in the ICU, hemodynamics continued to deteriorate, and the patient went into cardiac arrest. Cardiopulmonary resuscitation (CPR) was initiated, but return of spontaneous circulation (ROSC) could not be achieved after 14 cycles of CPR; subsequently, the patient was declared dead.

**Table 2:** Arterial blood gases pCO2: partial pressure of carbon dioxide; pO2: partial pressure of oxygen; HCO3: bicarbonate

Arterial blood gases	Normal range	Results
pH	7.35-7.45	7.09
pCO2	35-48	59
pO2	83-108	143
HCO3	18-23	18
Base excess	-2 to +3	-12

## Discussion

Pneumonectomy is a relatively uncommon surgical procedure. It is often considered for patients with advanced lung cancer not manageable with lung-sparing resection. Compared to other thoracic procedures, pneumonectomy has the highest morbidity, and its associated perioperative mortality ranges from 7% to 11% [16]. Morbidity and mortality related to post-pneumonectomy changes mainly depend on the duration of the onset, the frequency of respiratory tract infections, and cardiac arrhythmias. In addition, it all depends on the degree of the mediastinal shift and cardiac herniation. It is a rare complication, and a very limited number of cases have been reported worldwide.

Cardiac herniation is a lethal complication with a mortality rate of 100% if not recognized and managed on time. Even in cases where it is treated timely, the mortality rate remains around 50% [17]. Occurrence of cardiac herniation is mostly in the immediate postoperative period, with 75% of the cases occurring at the end of surgery and most within the first 24 hours [18].

The pathophysiology and clinical condition of the patient depend on the degree and side of cardiac herniation. This complication has also been reported to occur without any hemodynamic instability immediately. In left pneumonectomy, the mediastinum gets shifted to the right by the arrangement of the aortic arch in the sagittal plane, while in right pneumonectomy, left-sided displacement occurs with the aortic arch arranged in the frontal plane [19].

In right-sided cardiac herniation, the heart rotates clockwise (dextrorotation or dextrocardia) leading to the twisting of major vessels (superior vena cava and inferior vena cava), causing them to obstruct and thus leading to the rapid decrease in cardiac output.

The main clue for early recognition is the characteristic hemodynamic status. Understanding its pathophysiology as well as having the knowledge of the typical radiologic investigation is crucial for early diagnosis [20]. Under these circumstances, it should be noted that a hemodynamically unstable patient needs urgent re-thoracotomy and immediate repositioning of the herniated heart.

There have been many case reports of the late onset of signs and symptoms of cardiac herniation. Two of them showed hemodynamic instability occurring 24 hours after surgery, and one case report documented herniation at 14 weeks following surgery.

As per the previous literature, surgical correction of cardiac herniation should be done immediately as soon as the diagnosis is made. Immediate treatment includes restoration of hemodynamic stability. Before transferring to the OR, keep the patient in the lateral decubitus position, keeping the non-surgical side down, avoiding the hyperinflation of the lung. The only effective and definitive treatment is re-thoracotomy to reduce the hernia and pericardial defect repair.

The key to early management is the early recognition of the signs and symptoms. Hemodynamic instability, ECG changes, and echocardiography can be the early determinants of cardiac herniation. CT scan and magnetic resonance imaging (MRI) can easily diagnose or suggest the degree of mediastinal shift and cardiac herniation.

## Conclusions

We recommend that all patients who undergo extrapleural pneumonectomy be monitored closely. In case of hemodynamic collapse in the postoperative period, cardiac herniation should be considered among other differential diagnoses. ECG changes and echocardiography are also useful non-invasive tools to confirm the diagnosis.
